# Glutaredoxin2 reduces age-associated B cell differentiation through maintaining redox homeostasis

**DOI:** 10.3389/fphar.2025.1593816

**Published:** 2025-10-15

**Authors:** Yuan Jiang, Chunli Sun, Qilin He, Shujun Liu, Shihao Tian, Yan Zhang, Yatao Du, Fubin Li, Huihui Zhang

**Affiliations:** ^1^ Shanghai Institute of Immunology, Faculty of Basic Medicine, Key Laboratory of Cell Differentiation and Apoptosis of Chinese Ministry of Education, Shanghai Jiao Tong University School of Medicine, Shanghai, China; ^2^ Center for Immune-Related Diseases at Shanghai Institute of Immunology, Ruijin Hospital, Shanghai Jiao Tong University School of Medicine, Shanghai, China; ^3^ Ministry of Education-Shanghai Key Laboratory of Children’s Environmental Health, Xinhua Hospital, Shanghai Jiao Tong University School of Medicine, Shanghai, China

**Keywords:** Glutaredoxin2, reactive oxygen species (ROS), redox homeostasis, antioxidant, resistant diseases

## Abstract

**Background:**

The redox system plays a pivotal role in autoimmune diseases and cancer, with oxidative stress and antioxidant adaptations driving pathological processes. Age/autoimmunity-associated B cells (ABCs), characterized by elevated ROS levels, are implicated in autoimmune disorders such as systemic lupus erythematosus (SLE). However, the mechanisms linking ROS to ABC differentiation remain unclear. Glutaredoxin 2 (Grx2), a key oxidoreductase, regulates redox homeostasis, but its role in autoimmune B cell biology is underexplored.

**Methods:**

Using wild-type and Grx2-knockout mice, we examined ROS levels and ABC differentiation. In vitro, ABC differentiation was induced with IL-21 and TLR7 agonist, and the effect of the antioxidant N-Acetyl-L-Cysteine (NAC) was assessed. The SLE-prone ShipΔB model crossed with Grx2−/− mice was used to evaluate autoimmune pathology.

**Results:**

ABCs exhibited higher ROS levels than follicular B cells, and NAC reduced ABC differentiation rate by 50%, demonstrating ROS dependency. Grx2 deficiency amplified ROS levels and ABC proportions in aged mice, correlating with accelerated autoimmunity. In ShipΔB mice, Grx2 deletion exacerbated ABC differentiation, CD4+ T cell activation, and anti-dsDNA autoantibody titers.

**Conclusions:**

Grx2 acts as a redox checkpoint that limits ABC-driven autoimmunity by modulating ROS. The Grx2–ROS axis represents a potential therapeutic target for SLE and related chronic inflammatory diseases.

## 1 Introduction

The redox system has emerged as a critical pharmacological target in resistant diseases, particularly in oncology and autoimmunity, where oxidative stress and antioxidant adaptations drive pathological processes (Muri and Kopf, 2021; Jena et al., 2023; Halliwell, 2024). In recent years, an increasing number of studies have found that individuals in an autoimmune state are more likely to develop cancer, further suggesting that there may be a common pathological basis between autoimmunity and cancer development (Yang et al., 2025; Zhan et al., 2025; Han et al., 2021). Oxidative stress and related chronic inflammation may constitute an important component of this pathological basis (Elkoshi, 2022; Urbonaviciute et al., 2019; Mittal et al., 2013).

Age/autoimmunity-associated B cells (ABC) were first reported in 2011 (Hao et al., 2011; Rubtsov et al., 2011). These studies found a significant accumulation of a non-conventional B cell subset in aged female mice, characterized by CD11c^+^Tbet^+^. These cells accumulate with age, and appeared earlier in mice prone to autoimmunity, hence were named age/autoimmunity-associated B cells (ABC). Subsequent research revealed that ABC also significantly appears in viral infections and chronic inflammatory diseases represented by autoimmune diseases, including systemic lupus erythematosus (SLE), rheumatoid arthritis, Sjögren’s syndrome and multiple sclerosis, playing an important role (Cancro, 2020; Xie et al., 2025; Dai et al., 2024). The elevated levels of ABC in patients with these autoimmune diseases suggest their involvement in the pathogenesis of these conditions. Notably, ABCs harbor autoreactive antibody specificities and correlate with poor clinical responses, positioning them as therapeutic targets.

Endogenous ROS and the antioxidant system have been shown to participate in the proliferation and differentiation of B cells by maintaining a state of continuous activation (Zhang W. et al., 2019; Martinis et al., 2025). B cell receptor (BCR) and Toll-like receptor (TLR) stimulation can activate membrane-associated NADPH oxidase, leading to elevated ROS levels within B lymphocytes (Capasso et al., 2010; Bertolotti et al., 2016). ROS can further activate protein tyrosine kinases and inhibit protein tyrosine phosphatases, keeping B lymphocyte signaling in a state of continuous activation, thereby enhancing the strength and duration of B cell signals through positive feedback (Reth, 2002; Singh et al., 2005; Bertolotti and Sitia, 2018). The degree and duration of signal activation determine the differentiation of B lymphocytes into different subsets (Casola, 2007; Wheeler and Defranco, 2012). However, it is still unknown how ROS affects the differentiation of ABC and the underlying mechanisms involved.

Glutaredoxin 2 (Grx2), a thiol-disulfide oxidoreductase, is a key component of cellular antioxidant defense. By catalyzing glutathione-dependent protein deglutathionylation, Grx2 maintains redox homeostasis and protects against oxidative damage (Ogata et al., 2021; Zhang et al., 2014; Lillig, Berndt, and Holmgren, 2008). While Grx2’s roles in cancer, cardiac pathology and neurodegeneration are documented (Gellert et al., 2020; Kanaan et al., 2018; Lepka et al., 2017), its function in autoimmune regulation, particularly in B cell biology, remains unclear. Our previous work demonstrated that Grx2 loss in the cancerous HeLa cells reduces antioxidant capacity and viability (Zhang et al., 2014), suggesting its potential regulatory role in ROS-sensitive immune cells. Intriguingly, Grx2 deficiency exacerbates age-associated pathologies linked to oxidative stress (Lapenna, 2023), raising the possibility that Grx2 may restrain ABC differentiation by modulating ROS in autoimmune contexts.

To investigate this hypothesis, we employed the Grx2-knockout mouse model, and the ShipΔB mouse model, a B cell-specific Src homology 2-containing inositol 5-phosphatase 1 (SHIP-1) knockout strain that develops spontaneous SLE-like autoimmunity (O'Neill et al., 2011; Zhang H. et al., 2019). Here, we report that compared to follicular B cells, ABCs exhibit elevated ROS levels, which are essential for their differentiation. Grx2 deficiency further amplifies ROS production and ABC differentiation, aggravating T cell activation and autoantibody titers. These findings establish Grx2 as a novel redox checkpoint controlling ABC-driven autoimmunity and propose therapeutic targeting of the Grx2-ROS axis in SLE and related chronic inflammation based resistant diseases.

## 2 Materials and methods

### 2.1 Animals

Grx2 knockout mice were generated by the Central Institute for Experimental Medicine and Life Science (CIEM, https://www.ciea.or.jp), Kawasaki, Japan, by targeted deletion of the exons 1c, 1a and 2 of *Glrx2* (Grx2 coding) gene (Supplementary Figure S1). ShipΔB mice were generously provided by Professor Jeffrey Ravetch at Rockefeller University in New York, United States. Grx2−/−, ShipΔB mice were generated by crossing ShipΔB mice with Grx2−/− mice. C57BL/6 wild type mice and MRL/lpr mice were purchased from Shanghai Lingchang Biotechnology Co., Ltd. All animals were kept in the animal facility of Shanghai Jiaotong University School of Medicine (SJTUSM) Department of Laboratory Animal Science in specific pathogen-free (SPF) conditions, with experiments conducted using age- and sex-matched mice to ensure experimental consistency. All experiments were approved and conducted in compliance with institutional guidelines and had been approved by the SJTUSM Institutional Animal Care and Use Committee (IACUC).

### 2.2 Flow cytometry

Flow cytometry analyses were performed using BD FACSymphony™ A3 (BD Biosciences) or BD LSRFortessa™ X-20 analyzer (BD Biosciences). Cells were stained with anti-mouse/human B220 (RA3-6B2), anti-mouse CD19 (1D3), anti-mouse CD11c (N418) for ABC cell analysis, with anti-mouse CD4 (RM4-5), anti-mouse CD44 (IM7), anti-mouse CD62L (MEL-14) for T_EM_ cell and naïve T cell analysis. For ROS level detection, cells were incubated with 2,7-dichlorofluorescein diacetate (H2DCFDA) (abcam Cat#ab113851) for 30 min at 37 °C before subjected to flow cytometry analysis. All antibodies were purchased from BD Biosciences, Biolegend or Thermo Fisher.

### 2.3 B cell sorting

Mouse splenic follicular (FO) B cells were magnetically (MACS) sorted. Splenocytes were incubated with biotinylated anti-CD43 (2.5 μg/mL), anti-CD11c (2 μg/mL) and anti-GL7 (2 μg/mL) in MACS buffer (PBS, pH 7.2, 0.5% BSA, 2 mM EDTA) for 25 min, washed with MACS buffer, then incubated with anti-biotin microbeads (Miltenyi) for 20 min, followed by another washing with MACS buffer. Cells were then loaded on the LS column (Miltenyi), and the flow-through was collected as FO B cells.

### 2.4 *In vitro* induction of ABC differentiation

MACS-sorted FO B cells were cultured in RPMI1640 media (HyClone) containing 10% FBS (Gibco), 5 mg/mL sodium pyruvate (Gibco), 100 U/mL penicillin (Gibco), 0.1 mg/mL streptomycin (Gibco), 1X NEAA (Gibco), 1 μg/mL CL097 (Invivogen) and 25 ng/mL IL-21(Peprotech) in the presence or absence of 5 mM N-Acetyl-L-Cysteine (NAC) (Beyotime) for 48 h before flow cytometry analysis.

### 2.5 ELISA for Grx2 and anti-dsDNA autoantibody detection

The enzyme-linked immunosorbent assay (ELISA) for Grx2 was performed following a standardized sandwich protocol. Briefly, 96-well plates were coated with 2 μg/mL anti-mouse Grx2 (IMCO) in PBS, pH7.0 and incubated overnight at 4 °C. After washing with PBST (PBS containing 0.05% Tween-20), the plates were blocked with 1% BSA in PBS for 2 h. Serial dilutions of standard Grx2 protein and spleen homogenates were added to the wells and incubated for 2 h at 4 °C, followed by three PBST washes. Biotinylated anti-mouse Grx2 were added and incubated for 1 h at 4 °C, followed by four washes and standard horseradish peroxidase (HRP)-conjugated streptavidin incubation, washing, and TMB substrate reaction. Absorbance was measured at 650 nm using a microplate reader. Anti-dsDNA IgG antibodies were detected by ELISA kit (QUANTA Lite, 708510).

### 2.6 Protein glutathionylation status detection

Fresh mouse spleens were ground in pre-cooled PBS and passed through a 70-μm cell strainer to obtain splenocytes. The splenocytes were then lysed on ice for 30 min using RIPA lysis buffer containing 10 mM iodoacetamide (IAM). Protein concentration was determined using a BCA assay kit. A total of 30 μg protein per sample was loaded onto a non-reducing 10% Bis-Tris SDS-PAGE gel and separated by electrophoresis. The separated proteins were subsequently transferred onto a nitrocellulose membrane (Pall Corporation, United States). The membrane was blocked with 5% non-fat milk for 1 h at room temperature, followed by overnight incubation at 4 °C with gentle shaking in primary antibodies (anti-GSH, 1:2000 dilution, Abcam PLC., United Kingdom; anti-actin, 1:5000, Proteintech Inc., China). Subsequently, the membrane was incubated with an HRP-conjugated secondary antibody (1:1000 dilution, Beyotime Biotechnology, China) for 1 h at room temperature. Immune complexes were visualized using Immobilon Western HRP substrate (WBKLS500, Millipore, United States), and images were captured with a chemiluminescence imaging system (Bio-Rad Corporation, United States). Grayscale values of the bands were quantified with VisionWorks (Analytik Jena, Germany).

### 2.7 Statistical analysis

**FIGURE 1:**
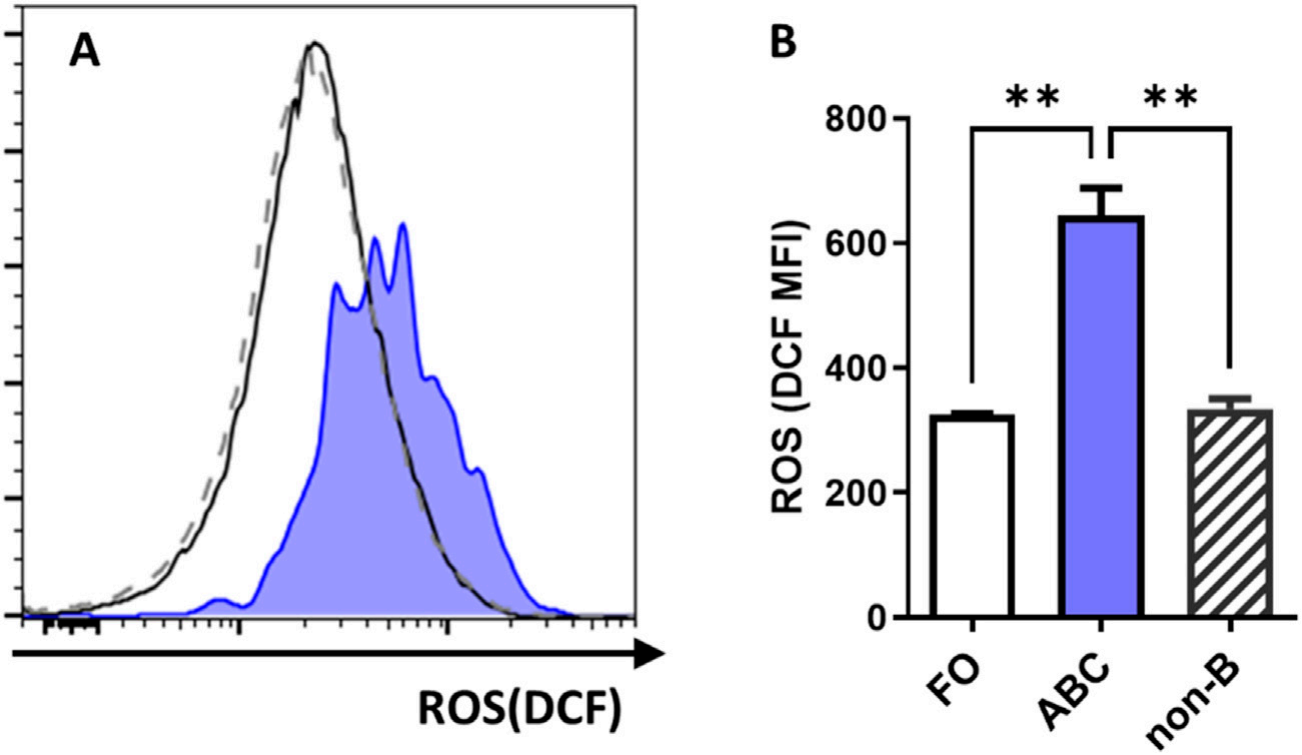
ROS levels in splenic B cell and non-B cell subsets of WT mice. **(A)** Representative histogram flow cytometry plot showing fluorescence signal intensity of ROS in follicular B cells (solid line), ABC (shaded), and non-B cells (dashed) from spleens of 6-month-old female wild type C57BL/6J mice. **(B)** Bar graph of ROS levels in the cell subsets (n = 2). FO, follicular B cells; non-B, non-B cells; MFI, mean fluorescence intensity; DCF, oxidized product of the ROS dye 2,7-dichlorofluorescein diacetate (H2DCFDA), ROS indicator. Data are represented as mean ± SEM. Ordinary one-way ANOVA was used in panel B **p < 0.01.

**FIGURE 2:**
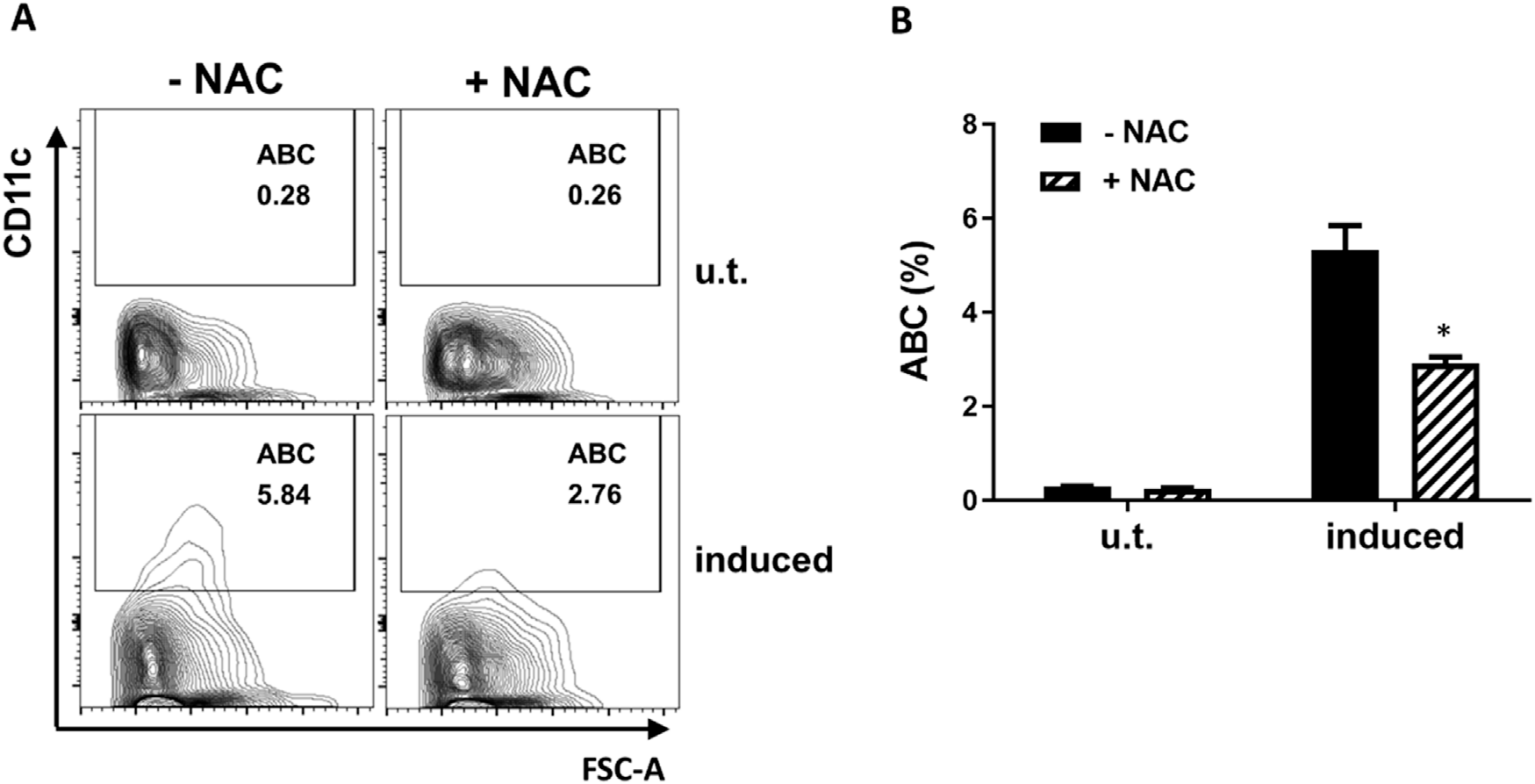
NAC inhibits the *in vitro* differentiation of ABCs. **(A)** Representative flow cytometry plot showing the percentage of ABC cells among FO B cells MACS isolated from wild type C57BL/6J mice and cultured for 48 h in the absence (u.t.) or presence (induced) of IL-21 and CL097. -NAC, culture in the absence of NAC; +NAC, in the presence of 5 mM NAC. **(B)** Bar graph of the ABC percentage of the cell cultures in panel **(A)** experiment (n = 2). Data are represented as mean ± SEM. Unpaired t-test was used in panel B *p < 0.05.

**FIGURE 3:**
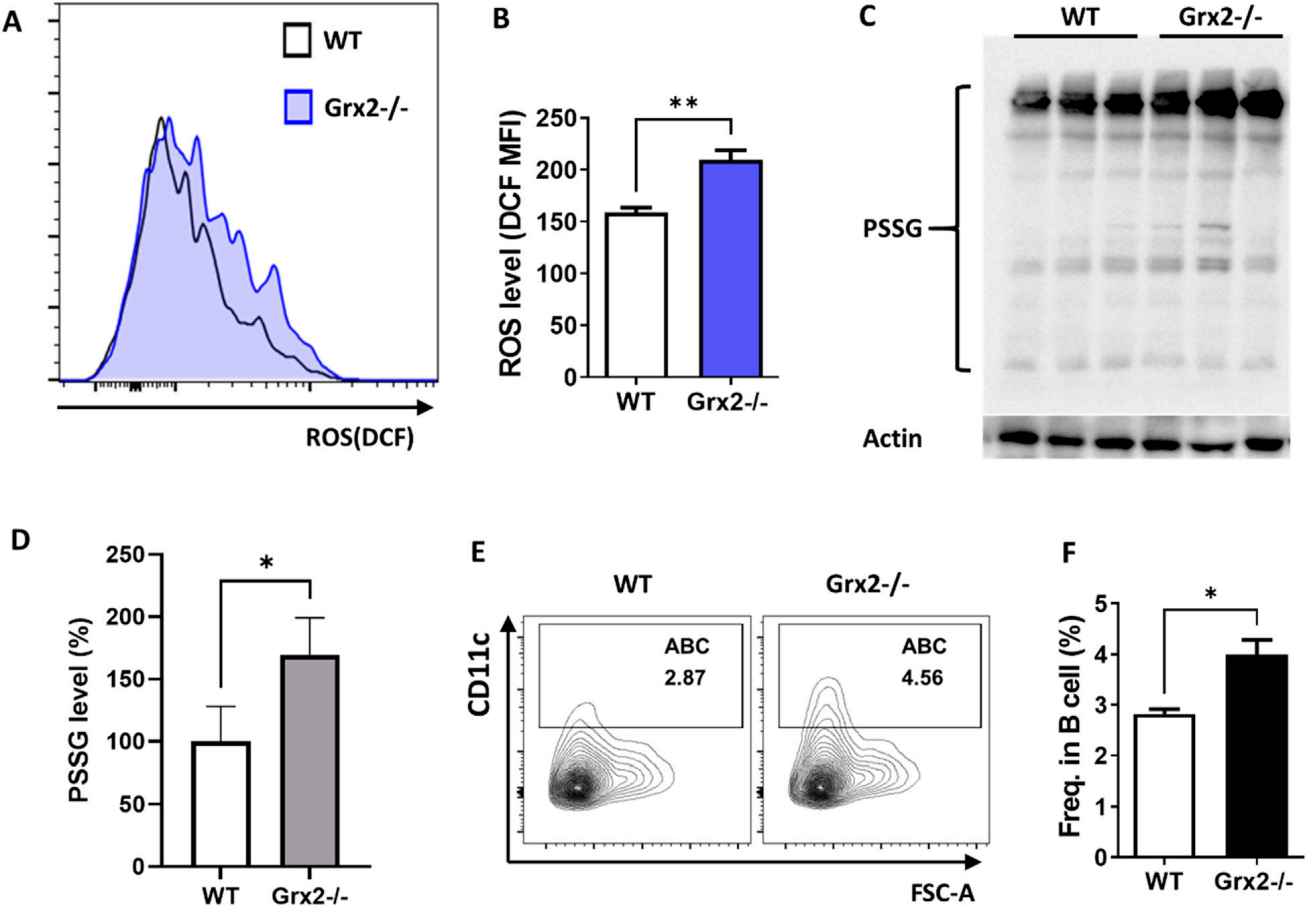
Aged Grx2 knockout mice have increased ABC differentiation. **(A)** Representative flow cytometry histogram plot showing ROS levels in splenic ABCs of 18-month-old female WT (solid line) and Grx2−/− (shaded) mice; **(B)** Bar graph of data from the panel A experiment (n = 3). **(C)** Protein glutathionylation level evaluated by Western blot after separation with non-reducing SDS-PAGE. Western blot of Actin was used as loading control. **(D)** Bar graph of PSSG levels represented by ratio of grayscale values of PSSG versus loading control for each sample in the panel C experiment, and the WT mean value was set as 100% (n = 3). **(E,F)** Representative flow cytometry profile **(E)** and bar graph **(F)** showing the percentage of ABC among splenic B lineage (B220^+^CD19^+^) cells of the aged WT and Grx2−/− mice (n = 3). Data are represented as mean ± SEM. Unpaired t-test was used in panel B, D and F. *p < 0.05, **p < 0.01.

**FIGURE 4:**
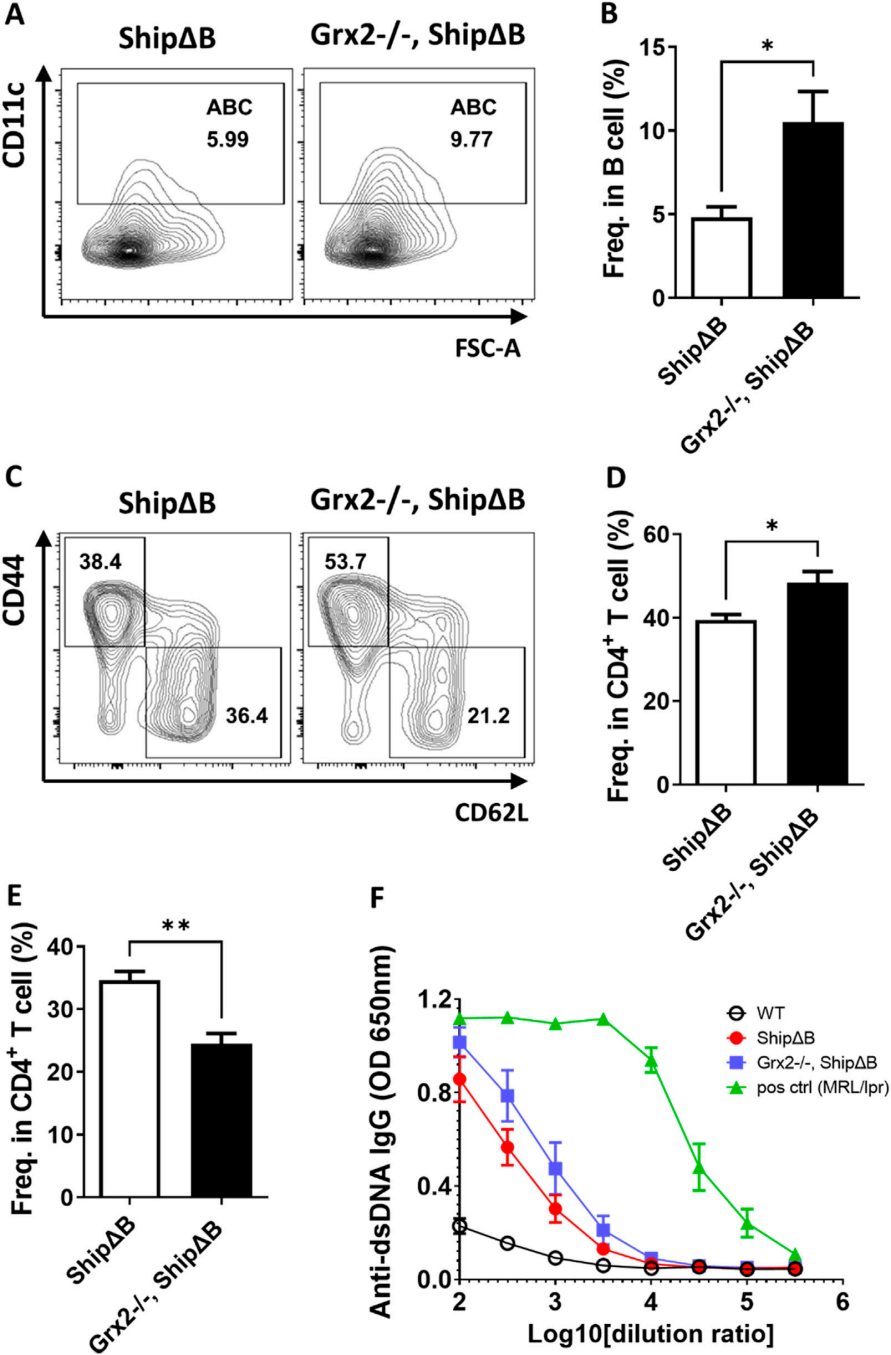
Grx2 knockout in ShipΔB mice exacerbates ABC differentiation and lupus phenotype. **(A,B)** Representative flow cytometry profile **(A)** and bar graph **(B)** showing the percentage of ABC among splenic B lineage (B220^+^CD19^+^) cells of ShipΔB and Grx2−/−, ShipΔB mice (5-month-old, male, n = 3). **(C–E)** Representative flow cytometry profile **(C)** and bar graph **(D,E)** showing the percentage of CD44^+^CD62L^−^ effector/memory cells (T_EM_) **(C,D)** and CD44^−^CD62L^+^ naïve cells **(C,E)** among CD4^+^ T cells in the spleen of these mice. **(F)** Anti-dsDNA IgG levels in the serum of the 5-month-old WT (black circle), ShipΔB (red dot), Grx2−/−, ShipΔB (blue square) and 7-month-old MRL/lpr (green triangle) mice. Data are represented as mean ± SEM. Unpaired t-test was used in panel B, D, and E *p < 0.05, **p < 0.01.

Statistical analyses were performed using GraphPad 9.0, and data were expressed as mean ± SEM. Ordinary one-way ANOVA was used in Figure 1, and unpaired t-test was used in Figures 2–4. A p-value <0.05 was considered significant (*p < 0.05, **p < 0.01).

## 3 Results

### 3.1 ABCs have higher ROS levels compared to follicular B cells and depend on ROS for effective differentiation

ABCs are primarily generated from naive follicular B cells (Cancro, 2020). Thus, we first measured and compared the ROS levels in ABCs and naive FO B cells from wild type mice. Flow cytometric analysis revealed that in the spleens of 6-month-old wild type mice, the ROS levels in ABC were significantly higher than those in FO B cells and other non-B cells (Figure 1). These results suggest that elevated ROS levels are a characteristic feature distinguishing ABC from resting naive FO B cells. However, it is not yet known if the elevated ROS plays a promoting or inhibitory role in the differentiation of FO B cells into ABC.

To explore the role of ROS in the differentiation of FO B cells into ABC, we utilized an *in vitro* system for inducing ABC differentiation from Naradikian et al.’s and our previous studies (Naradikian et al., 2016; Zhang H. et al., 2019). Purified splenic FO B cells were cultured in the presence of IL-21 and CL097 (a TLR7/8 agonist), which primed these B cells to express CD11c and differentiate into ABC. The antioxidant N-Acetyl-L-Cysteine (NAC) was also included in the culture system in a part of the samples to reduce the ROS levels in these cells. As shown in Figure 2, NAC treatment decreased the proportion of follicular B cells differentiating into ABC by approximately 50%. These results suggest that the differentiation of follicular naïve B cells into ABC requires physiological levels of ROS.

### 3.2 Aged Grx2 knockout mice have increased ABC differentiation

In our previous research, we found that Grx2 has important antioxidant functions, shown by the diminished antioxidant capacity and cell viability of cervical cancer HeLa cells with reduced Grx2 expression (Zhang et al., 2014). To investigate the role of Grx2 and its antioxidant effect in the differentiation process of ABC, we applied Grx2 knockout mice. As shown in Supplementary Figure S1, exons 1c, 1a, which contain the start codon for Grx2, and exon 2, were deleted, preventing the expression of the Grx2 protein. We identified the Grx2 knockout at the DNA level using PCR (Supplementary Figure S2). Additionally, we measured the expression levels of Grx2 in the spleen tissue of the mice using ELISA, it was found that the Grx2 levels in heterozygous Grx2 ± mice were reduced by 50% compared to wild type (WT) mice, while homozygous Grx2−/− mice completely lost Grx2 expression, confirming the knockout of Grx2 at the protein level (Supplementary Figure S3). Grx2−/− mice and age- and sex-matched WT C57BL6/J mice were kept until they were 18 months old. Using flow cytometry, we assessed the levels of ABC cell differentiation and ROS levels in the spleens of these mice. We found that the ROS levels in ABCs from Grx2−/− mice were significantly higher than those in ABCs from WT mice (Figures 3A,B). As Grx2 plays a critical role in protein de/glutathionylation, we examined the effect of Grx2 knockout on protein glutathionylation (PSSG) levels in the splenocytes of these mice. As shown in Figures 3C,D, the PSSG level significantly increased in the Grx2−/− mice. Additionally, the proportion of ABC in total B cells in Grx2−/− mice was significantly greater than that in WT mice (Figures 3E,F). These results suggest that Grx2 may inhibit the differentiation of ABC by controlling and reducing ROS levels, and maintaining redox homeostasis.

### 3.3 Grx2 knockout in the ShipΔB lupus model aggravates ABC differentiation and exacerbates lupus disease activity

To further validate the above effects of Grx2, we crossed Grx2 knockout mice with a B cell intrinsic systemic lupus erythematosus (SLE) model (ShipΔB) to obtain Grx2−/−, ShipΔB mice. We then examined the SLE disease levels in these mice compared to age- and sex-matched ShipΔB mice. As shown in Figures 4A,B, the level of ABC differentiation in Grx2−/−, ShipΔB mice was significantly higher than that in ShipΔB mice. Grx2−/−, ShipΔB mice also exhibited higher activation levels of CD4^+^ T cells, as shown by a greater proportion of effector/memory CD4^+^ T cells (CD4^+^ T_EM_) compared to ShipΔB mice (Figures 4C,D), while the proportion of naive CD4^+^ T cells was lower than that in ShipΔB mice (Figures 4C,E). We used ELISA to measure the levels of anti-dsDNA autoantibodies in the serum of these mice and found that the levels were elevated in Grx2−/−, ShipΔB mice compared to ShipΔB mice (Figure 4F). These results further indicate that Grx2 can control ROS levels through its antioxidant activity, maintaining redox homeostasis, thereby reducing ABC differentiation, decreasing T cell activation, lowering autoantibody production, and modulating the progression of SLE.

### 3.4 Human SLE patients exhibit lower GLRX2 expression

**FIGURE 5:**
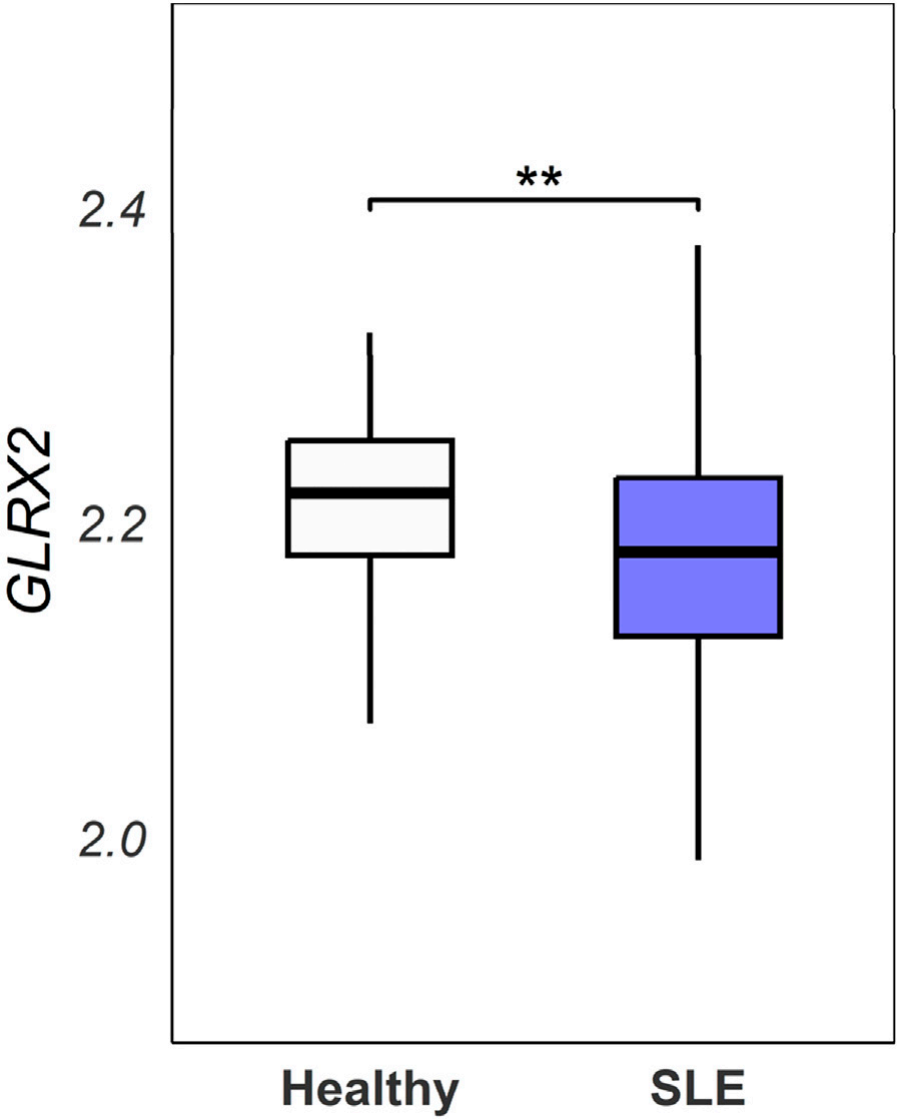
Analysis of the GSE65391 dataset. GLRX2 expression levels (log2-transformed normalized values) were compared between SLE patients (n = 923) and healthy controls (n = 48) from the GSE65391 dataset. Expression values represent the mean of three probes (ILMN_1680727, ILMN_2258268, ILMN_1737912). Statistical analysis was performed using a linear model approach with empirical Bayes moderation (limma package), followed by Welch’s t-test (**p = 0.00814).

To explore the clinical relevance of the findings in this study, we performed analysis of a publicly available SLE dataset, the GSE65391 dataset (n = 923 SLE patients vs. 48 healthy controls). The analysis revealed significantly lower GLRX2 gene expression in SLE patients (p = 0.00814), as shown in Figure 5. This clinical correlation provides compelling support for our findings in the mouse lupus (ShipΔB) model.

## 4 Discussion

This study establishes a pivotal role for Grx2 in restraining ABC differentiation by modulating ROS homeostasis. Using Grx2−/− and ShipΔB mouse models, we demonstrated that ABCs exhibit elevated ROS levels compared to follicular B cells, a feature critical for their differentiation. Grx2 deficiency exacerbated ROS accumulation, amplified ABC differentiation, and aggravated SLE-like autoimmunity. These findings position Grx2 as a redox checkpoint that curbs ABC-driven autoimmunity, highlighting its therapeutic potential in ROS-dependent inflammatory diseases.

Our work bridges redox biology and autoimmune pathogenesis by elucidating how Grx2 regulates ABC differentiation through ROS modulation. The elevated ROS levels in ABCs align with prior studies showing that oxidative stress drives B cell hyperactivity via protein tyrosine kinase activation and phosphatase inhibition (Bertolotti and Sitia, 2018; Takano, Sada, and Yamamura, 2002). We speculate that Grx2, working as a thiol-disulfide oxidoreductase, counteracts this ROS induced B cell overactivation by deglutathionylating redox-sensitive targets, thereby maintaining cellular antioxidant homeostasis. This mechanism mirrors Grx2’s role in mitigating age-related pathologies, such as cancer incidence (Brzozowa-Zasada et al., 2024), cataract formation (Wu et al., 2014), or neurodegeneration (Wen et al., 2020), where Grx2’s deficiency accelerates oxidative damage or cell remodeling that may result in abnormal activation or carcinogenesis. Our findings extend Grx2’s protective function to autoimmune regulation, suggesting a conserved antioxidant defense axis across diverse pathologies.

Our findings on Grx2-regulated ABC differentiation are strongly supported by clinical observations in SLE patients. Faustini et al. reported that ABCs (characterized as IgD^−^CD27^−^CD11c^+^CD21^−^ or T-bet^+^CD11c^+^ subsets) demonstrate significant peripheral expansion that decrease post-rituximab treatment (Faustini et al., 2022). Jenks et al. identified a pathogenic DN2 subset (CD11c^+^CXCR5^−^CD21^−^) linked to anti-Smith/RNP antibodies, driven by TLR7 activation - a pathway consistent with our mouse model (Jenks et al., 2018). Tissue infiltration is evidenced by Arazi et al. through single-cell RNA-seq showing ABC enrichment in lupus nephritis kidneys, where these cells express antigen-presentation genes (HLA-DR, CD86), mirroring our findings of ABC-mediated T cell activation (Arazi et al., 2019). The GSE65391 dataset (Banchereau et al., 2016) demonstrates significantly reduced GLRX2 expression in SLE patients compared to healthy controls (p = 0.00814), establishing clinical relevance for our investigation of Grx2-mediated redox regulation. This finding aligns with well-characterized redox imbalances in SLE, where mitochondrial dysfunction drives excessive ROS production (Gergely et al., 2002) and compromised antioxidant defenses are observed (Perl, 2013). Importantly, Henning et al. (Henning et al., 2025) provided direct clinical evidence linking ABC accumulation to oxidative stress through positive correlations between ABC frequency and myeloperoxidase-DNA complexes, thereby bridging our mechanistic findings with human SLE pathology. These clinical observations collectively underscore the potential of targeting the Grx2-ROS axis in SLE. The conserved nature of ABCs across species and their redox-sensitive differentiation suggest novel therapeutic strategies could emerge from further exploration of this pathway.

While Grx2 promotes cancer cell survival under oxidative stress, its deficiency in this study exacerbates autoimmune pathology, suggesting context-dependent roles in redox regulation. Specifically, for activated ABCs or already malignantly transformed cancer cells, Grx2 can similarly provide antioxidant protection, especially in cell types sensitive to oxidative stress or when using anti-cancer drugs that induce oxidative stress. In these cases, Grx2 may instead promote disease progression, as suggested in previous studies (Lillig et al., 2004; Zhang et al., 2014; Gellert et al., 2020). Based on this information and the results of our study, we speculate that ABCs have a lower sensitivity to oxidative stress induced by endogenous ROS. Therefore, the mild increase in ROS caused by Grx2 deficiency primarily leads to excessive differentiation of ABCs without increasing their mortality. This is also consistent with reports that follicular B cells have more potent antioxidant capacities compared to T lymphocytes (Muri et al., 2019). We will further validate this hypothesis in future studies to clarify the effects of ROS of different sources on the differentiation, proliferation, and survival of ABCs.

Our findings indicate that Grx2 plays a critical role in regulating ABC differentiation through its antioxidant (specifically deglutathionylation) activity. While the exact molecular pathways remain to be fully elucidated, and identifying the key target protein(s) that undergo specific glutathionylation upon Grx2 deletion in these cells is an important direction for future research, several redox-sensitive signaling pathways emerge as likely mediators of this process based on existing literature. The NF-κB pathway represents a prime candidate, as it is known to be regulated by glutathionylation status (Dalle-Donne et al., 2009). Studies have shown that NF-κB activation in B cells is highly sensitive to oxidative stress, with glutathionylation of key cysteine residues in IKKβ inhibiting its activity (Reynaert et al., 2004). This is particularly relevant given NF-κB’s established role in B cell differentiation and survival (De Silva and Klein, 2015). STAT3 signaling also emerges as a potential mechanistic link between Grx2 and ABC differentiation. As demonstrated by Ding et al., STAT3 signaling is crucial for promoting B cell survival and differentiation (Ding et al., 2016). The STAT3 pathway is known to be redox-regulated, with glutathionylation of critical cysteines modulating its phosphorylation and nuclear translocation (Butturini et al., 2014). The mTOR pathway represents another redox-sensitive regulator of B cell fate. Franchina et al. has proved that glutathione-dependent redox control modulates mTORC1 activation and metabolic reprogramming in follicular B cells, highlighting subset-specific redox sensitivity in B cell immunity via mTOR signaling (Franchina et al., 2022). Given that Grx2 maintains cellular redox homeostasis, its deficiency could lead to mTOR pathway dysregulation through persistent oxidation of regulatory cysteine residues in mTOR complex components. The deficiency of Grx2 in ABC differentiation may therefore reflect its position as a regulator of multiple intersecting pathways that collectively govern B cell fate decisions. Future studies employing cell-specific knockout models will be needed to precisely delineate the relative contributions of these pathways to the Grx2-ABC axis.

While our study demonstrates that Grx2 deficiency elevates ROS levels and enhances ABC differentiation, we acknowledge the potential for compensatory upregulation of other antioxidant systems (e.g., glutathione peroxidase/glutathione reductase or thioredoxin pathways) to partially compensate for Grx2 loss to maintain redox homeostasis. Such compensatory mechanisms represent a well-established feature of redox biology, where antioxidant systems often exhibit functional redundancy to protect against oxidative stress (Muri et al., 2019; Halliwell, 2024). Notably, Grx2 exhibits cross-talk with the thioredoxin system, as it can reduce both Trx1 and Trx2 to protect cells from oxidative stress-induced apoptosis (Zhang et al., 2014). However, our data in this study indirectly imply that these compensatory mechanisms are insufficient to fully normalize ROS levels or ABC differentiation in Grx2-deficient mice, implying cell-type-specific dependencies on Grx2 for redox homeostasis. However, it remains a hypothesis pending direct experimental validation. Specifically, our study does not provide functional data on the activity of the Trx or other compensatory systems, which is essential to unequivocally confirm their inability to compensate in this setting. Thus, a key future direction will be to directly quantify the activity and adaptive response of other redox systems in this model, which will definitively test the compensatory hypothesis proposed here.

In summary, Grx2 is identified as a critical restrainer of ABC differentiation and autoimmune progression by tempering ROS-driven B cell activation. This study not only advances our understanding of redox regulation in autoimmunity but also provides a new pathway for developing targeted therapies aimed at mitigating diseases where oxidative stress fuels pathology, from SLE to age-related disorders.

## Data Availability

The datasets presented in this article are not readily available because The raw data supporting the conclusions of this article will be made available by the authors, without undue reservation. Requests to access the datasets should be directed to Huihui Zhang, huizha@sjtu.edu.cn.
