# Supplementary material for: Glutaredoxin2 reduces age-associated B cell differentiation through maintaining redox homeostasis
Source: Front Pharmacol. 2025 Oct 15;16:1593816. doi: 10.3389/fphar.2025.1593816 (PMC12568657; doi:10.3389/fphar.2025.1593816)
Supplement: Supplementary file 1 [file Supplementaryfile1.docx]

Supplementary Material


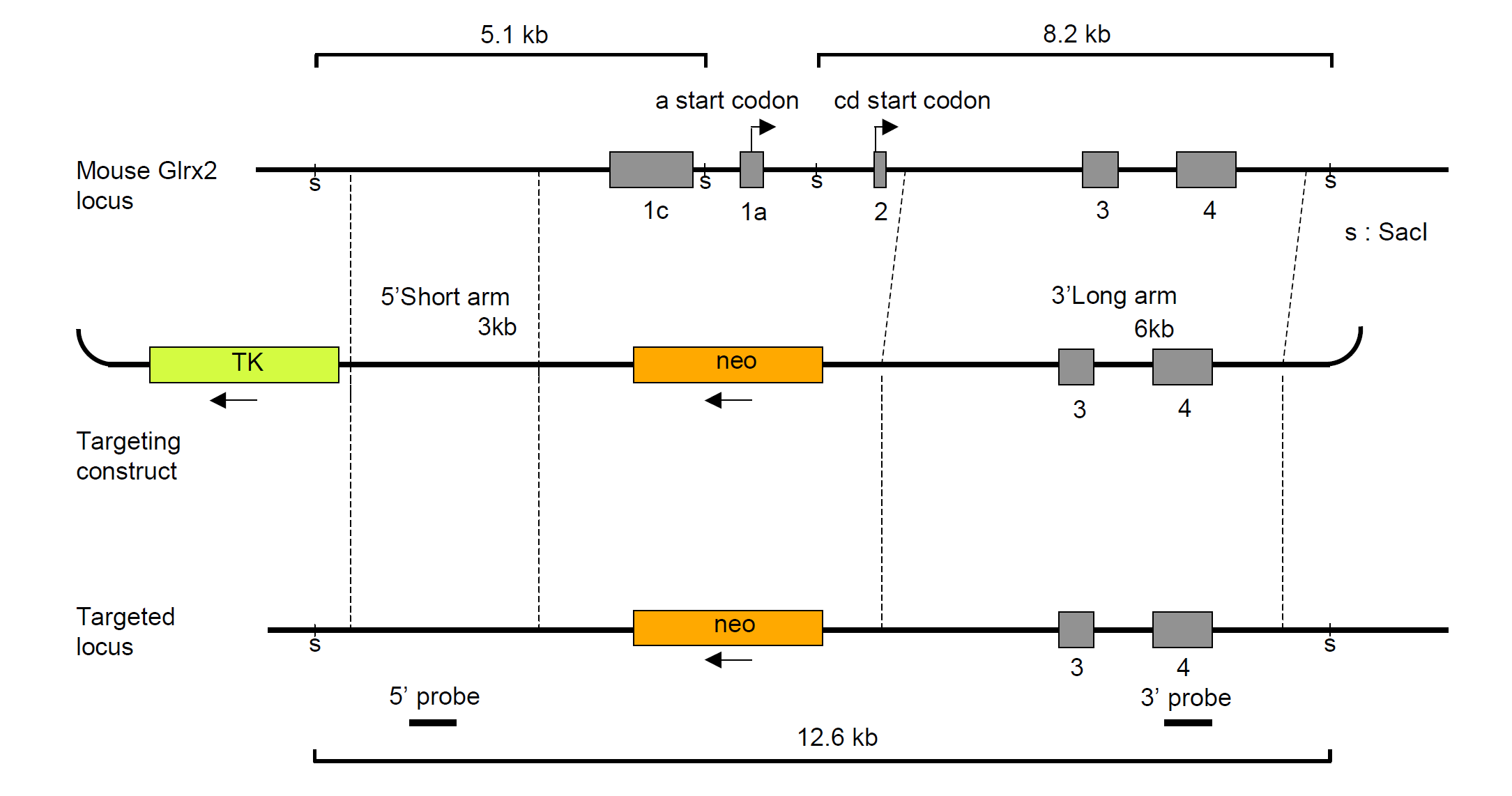


**Figure S1** Targeted disruption of the mouse *Glrx2* (Grx2 coding) gene. genomic structure of the mouse *Glrx2* locus (top), the targeting construct (middle), and the targeted locus (bottom) are shown.


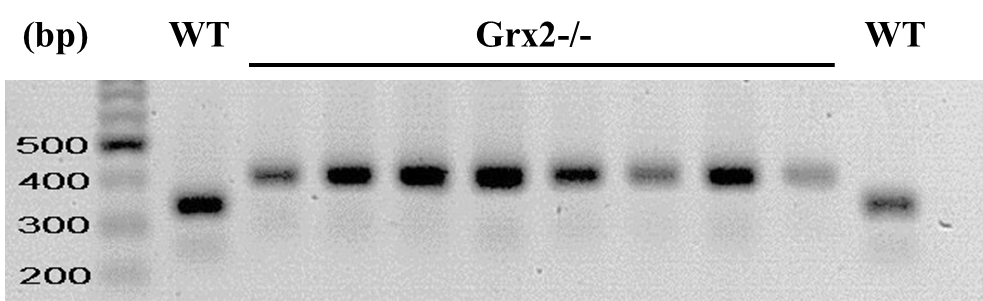


**Figure S2** PCR genotyping of Grx2 knockout (Grx2-/-) mice.


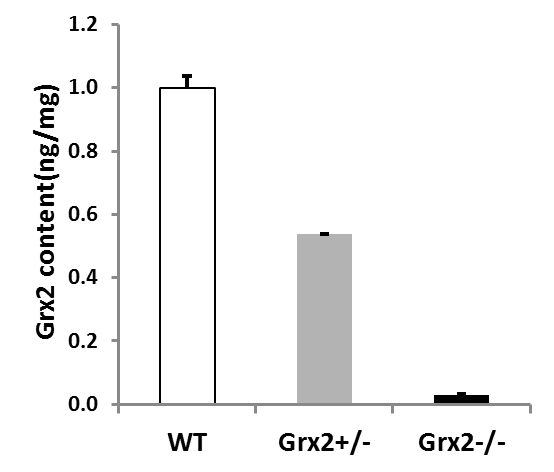


**Figure S3** ELISA Identification of Grx2 Levels in Mouse Spleen (2-month-old female, n=2).


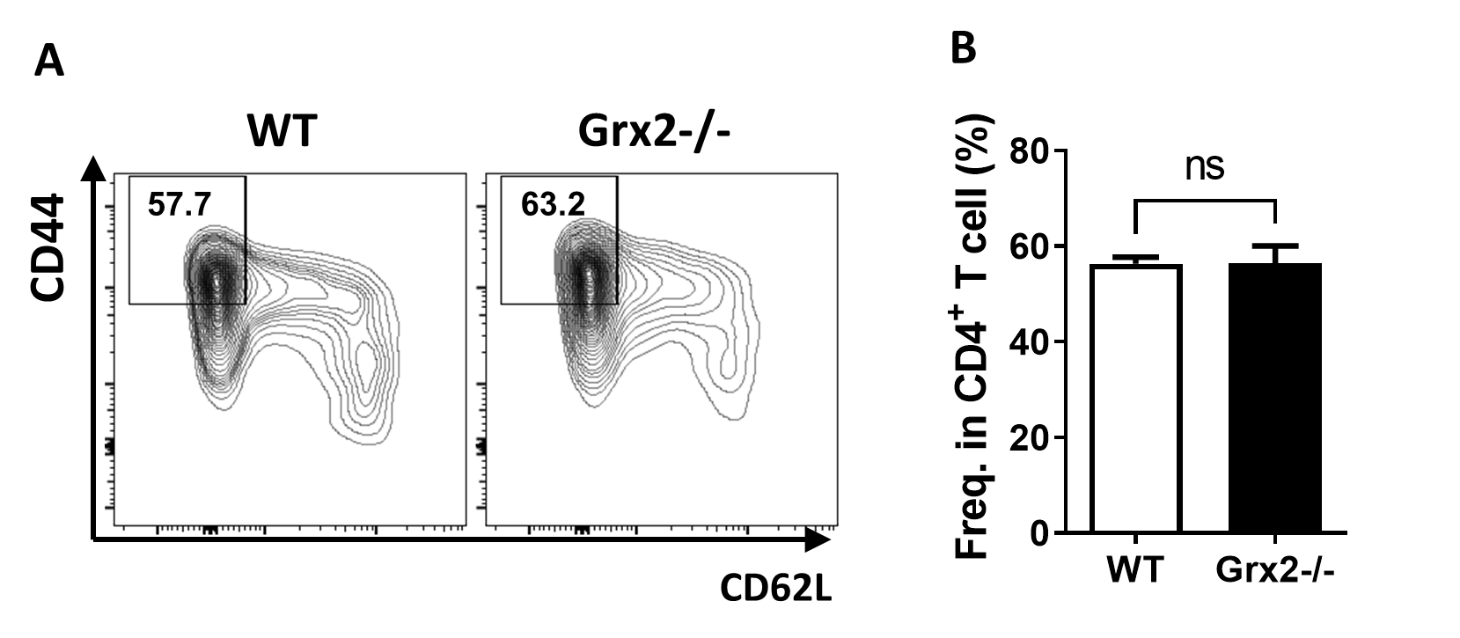


**Figure S4** Grx2-/- mice have similar CD4^+^ T cell activation profile comparing to age- and sex-matched WT mice. **(A and B)** Representative flow cytometry profile (A) and bar graph (B) showing the percentage of CD44^+^CD62L^−^ effector/memory cells (T_EM_) among CD4^+^ T cells in the spleen of these mice (18-month-old, female, n=3).
